# Description of long-term climate data in Eastern and Southeastern Ethiopia

**DOI:** 10.1016/j.dib.2017.03.025

**Published:** 2017-03-18

**Authors:** Messay Mulugeta, Degefa Tolossa, Gezahegn Abebe

**Affiliations:** a College of Development Studies, Addis Ababa University, Ethiopa; b Department of Sociology and Human Geography, University of Oslo, Norway

**Keywords:** Rainfall variability, Drought years, Seasonal precipitation concentration, Temperature, Ethiopia

## Abstract

This article presents long-term analyzed climate data from nine weather stations in eastern and southeastern parts of Ethiopia. At the outset of this data process, unrefined meteorological data was obtained from National Meteorological Agency (NMA) of Ethiopia for the analysis. The analyzed data in this article shows patterns of rainfall variability, frequency of drought years, seasonal concentration of precipitation and temperature conditions. As issues related to climate conditions are very intricate, different techniques and indices were applied to analyze and refine the data. The analysis reveals that eastern and southeastern parts Ethiopia are severely affected by recurrent droughts, erratic rainfall, and high and increasing temperature conditions. The long-term (1981–2009) mean annual total rainfall had been fluctuating between about 850 mm and 1350 mm. Most stations receive maximum rainfall in summer (June, July and August) except Gode which gets over 50% of its rainfall in spring season (March, April and May). The inter-annual rainfall difference was found to be very high. The Precipitation Concentration Index (PCI) is greater than 11 for all the stations showing that rainfall is concentrated in a few months. PCI is extremely high (greater than 20) for very dry stations such as Gode. Food production and consumer price index were found to be fluctuating with rainfall patters.

**Specification Table**

**Table:** 

Subject area	Environmental Studies
More specific subject area	Climate Change
Type of data	Figure and table
How data was acquired	Unprocessed secondary data
Data format	Analyzed
Data source location	Adama; Arba Minch; Ciro; Dire-Dawa; Gode; Jigjiga; Moyale; Yabello and Ziway meteorological stations.
Experimental factors	Data were obtained from the NMA and CSA of Ethiopia.
Experimental features	Computational analysis: Precipitation concentration indexes (PCI), percentages, averages, total values and graphic trend analysis were computed by using SPSS version 20 and Microsoft Excel softwares.
Data accessibility	The data is with this article.

**Data value**

• Gives picture on the changing environment in Ethiopia.
• Provides information on the patterns of rainfall variability, frequency of drought years, seasonal concentration of precipitation and temperature conditions in Ethiopia.
• Can be used to identify areas vulnerable to climate change for various forms of interventions.
• Useful to researchers and experts working in climate change, disaster risk management, food security and other related fields.

## 1 Data

**Fig. 1:**
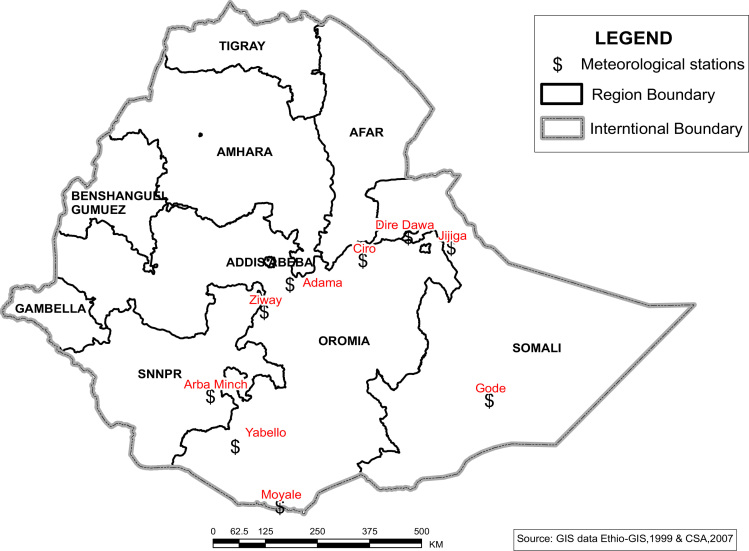
Location of sample weather stations in eastern, southern and southeastern Ethiopia.

**Fig. 2:**
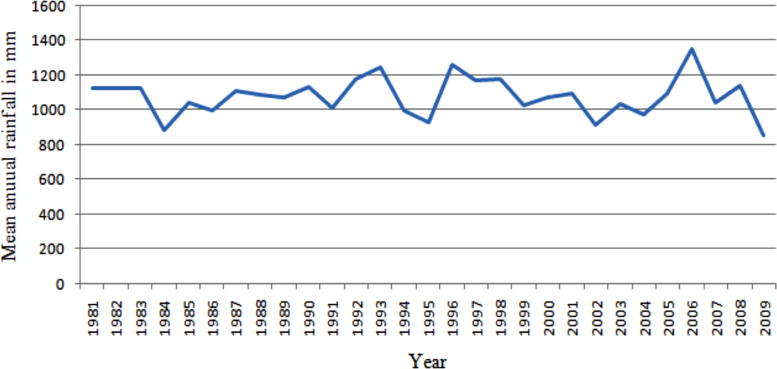
Long-term mean annual total rainfall in Ethiopia /1981–2009/.*Source*: Computed based on raw data from NMA of Ethiopia.

**Fig. 3:**
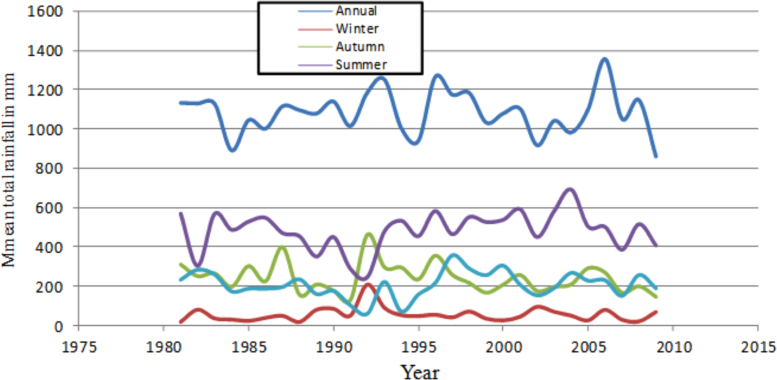
Long-term mean annual total and seasonal rainfall variability (1981–2009).*Source*: Computed based on raw data from NMA of Ethiopia.

**Fig. 4:**
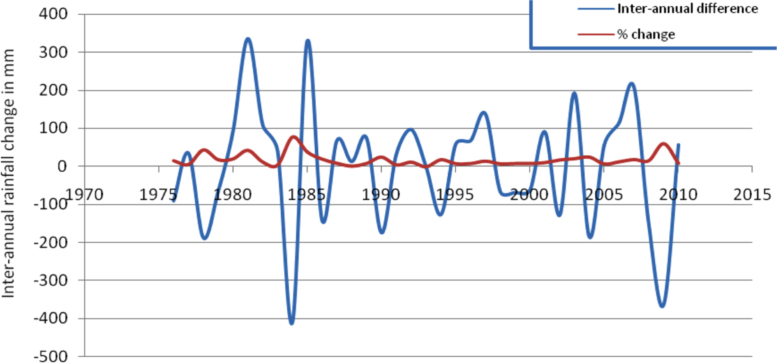
Inter-annual rainfall differences in southern and southeastern Ethiopia.*Source*: Computed based on raw data from NMA of Ethiopia.

**Fig. 5:**
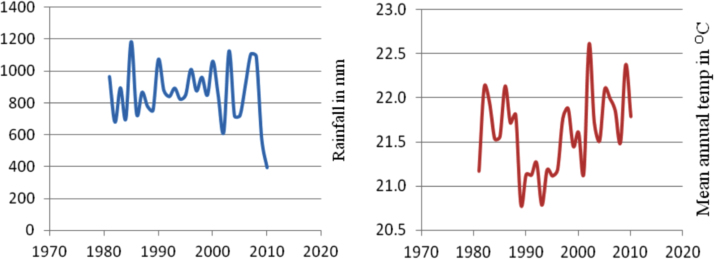
Rainfall and temperature patterns of Adama Weather Station.*Source*: Computed based on raw data from NMA of Ethiopia.

**Fig. 6:**
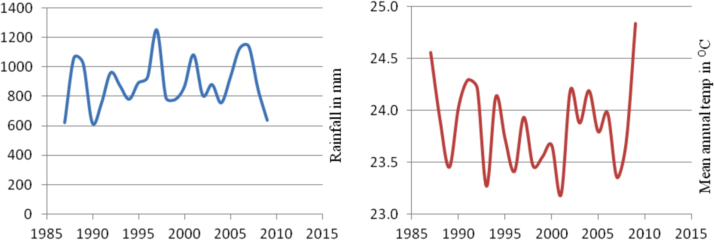
Rainfall and temperature patterns of Arba-Minch Weather Station.*Source*: Computed based on raw data from NMA of Ethiopia.

**Fig. 7:**
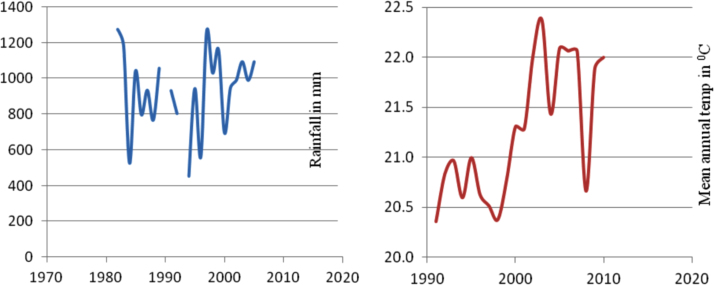
Rainfall and temperature patterns of Ciro Weather Station.*Source*: Computed based on raw data from NMA of Ethiopia.

**Fig. 8:**
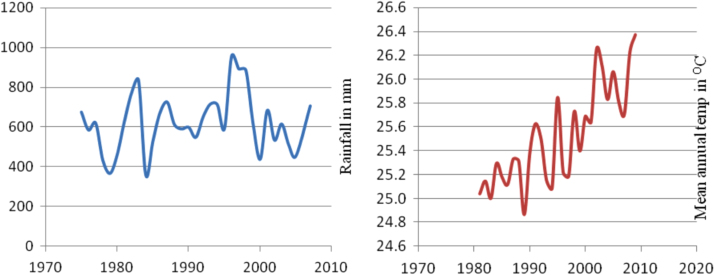
Rainfall and temperature patterns of Dire-Dawa Weather Station.*Source*: Computed based on raw data from NMA of Ethiopia.

**Fig. 9:**
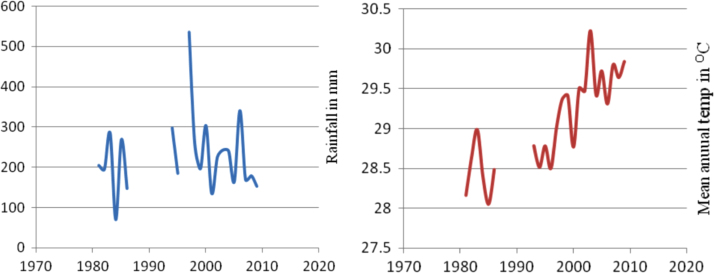
Rainfall and temperature patterns of Gode Weather Station.*Source*: Computed based on raw data from NMA of Ethiopia.

**Fig. 10:**
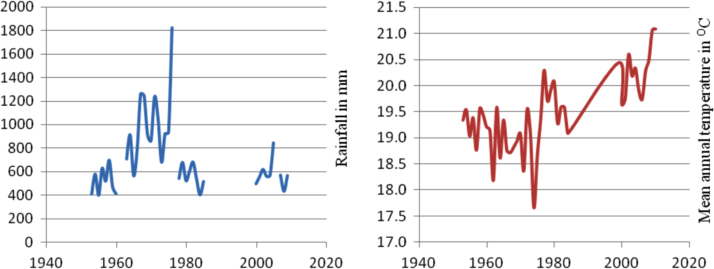
Rainfall and temperature patterns of Jigjiga Weather Station.*Source*: Computed based on raw data from NMA of Ethiopia.

**Fig. 11:**
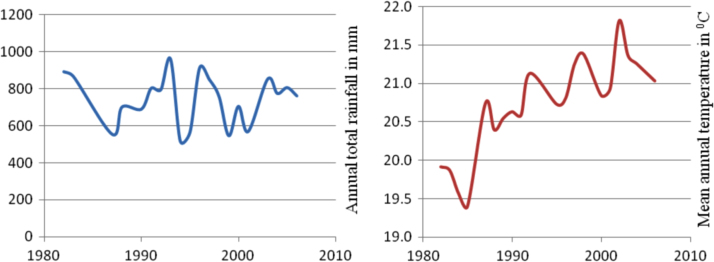
Rainfall and temperature patterns of Ziway Weather Station. *Note*: Both the rainfall and temperature data obtain from Moyale and Yabello Weather stations is highly discontinuous to present the trends of weather elements in graphs.*Source*: Computed based on raw data from NMA of Ethiopia.

The figures and tables in this article show analyzed data gathered from the nine weather stations across eastern and southeastern part of Ethiopia. Fig. 1 shows the location of the sources of data *(i.e*. sample meteorological stations) in Ethiopia. Data related to long-term annual total rainfall **(**Fig. 2**)** and mean annual total rainfall and seasonal variability in Ethiopia (Fig. 3) are presented. Fig. 4 demonstrates the inter-annual rainfall differences in southern and southeastern Ethiopia. The data in this figure shows that the mean total annual rainfall fluctuates highly. The inter-annual coefficient of variation is 20% implying severe moisture scarcity and substantial inter-annual rainfall variability. Fig. 5, Fig. 6, Fig. 7, Fig. 8, Fig. 9, Fig. 10, Fig. 11 show rainfall and temperature patterns of the sample stations. Rainfall variability is higher at drier stations (such as Gode and Dire Dawa) than wetter stations like Arba Minch and Adama. The inter-winter coefficient of variation is computed to be 0.69, while the variation between autumns, summers and springs is 0.31, 0.20 and 1.61, respectively. Following this the analysis of long-term annual rainfall and temperature conditions of Adama Weather Station (Fig. 6), Arba-Minch (Fig. 6), Ciro (Fig. 7), Dire-Dawa (Fig. 8), Gode (Fig. 9), Jigjiga (Fig. 10), and Ziway (Fig. 11) are presented. The graphs and tables farther show years of maximum and minimum rainfall or drought years across the areas. The trends of the spring (*belg*) rains of the weather stations (Fig. 11**A–H**) mean seasonal rainfall values of the stations (Fig. 13) and trend of temperature conditions (Fig. 14) in Ethiopia are shown. Table 1 shows the sample weather stations by PCI (Precipitation Concentration Index). Long-term mean annual rainfall and temperature values of the stations and long-term mean seasonal values of the stations are presented in Table 2, Table 3 respectively. Finally, in Fig. 15, Fig. 16 and 17 the association between total annual rainfall and food production, the intricacies between rainfalls and food gap and consumption price index are presented, respectively.

**Fig. 12:**
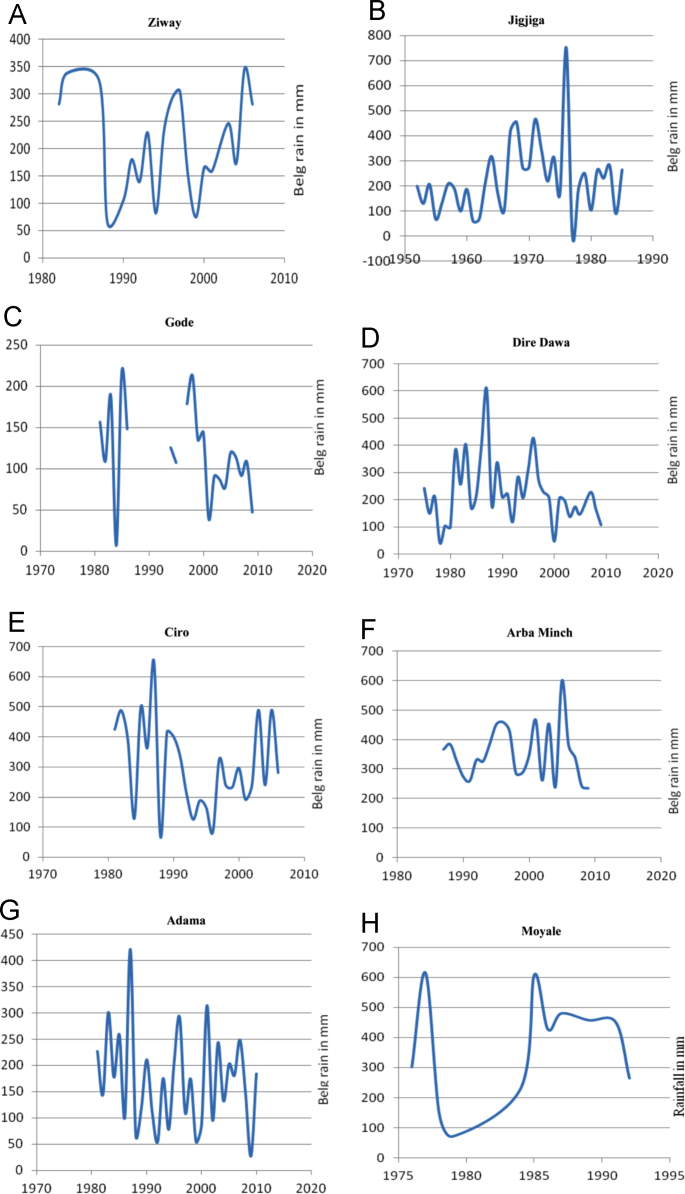
**(A–H)**: Analysis of trends of spring (*belg*) rains of the stations.*Source*: Computed based on raw data from NMA of Ethiopia.

**Fig. 13:**
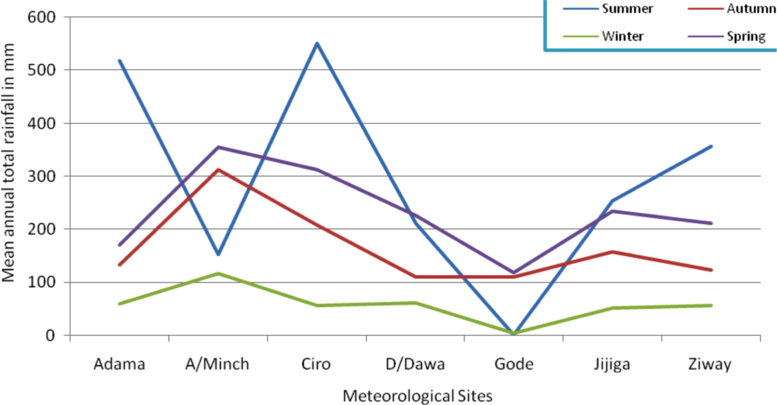
Long-term mean seasonal rainfall values of the stations.*Source*: Computed based on raw data from NMA of Ethiopia.

**Fig. 14:**
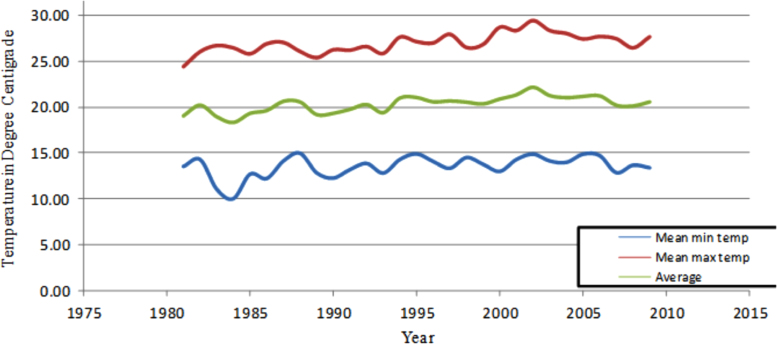
Long-term trend of temperature conditions in Ethiopia.*Source*: Computed based on raw data from NMA of Ethiopia.

**Fig. 15:**
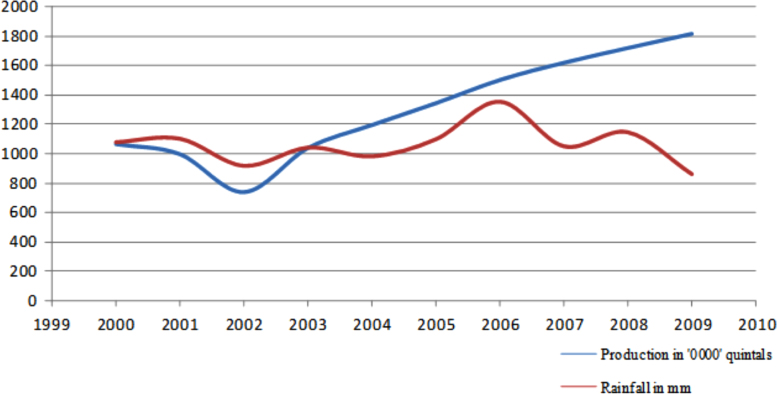
Association between total annual rainfall and food production. Note: Although the mean annual rainfall has been fluctuating, the overall food production and availability have increased substantially in the last 20 years [2]. An explanation to this is that farmer׳s farm inputs application and enhancing farmers’ knowledge. However, there is regional and local level droughts that adversely affect production, which made farmers vulnerable to food shortage.*Source*: Computed based on raw data from NMA and CSA of Ethiopia.

**Fig. 16:**
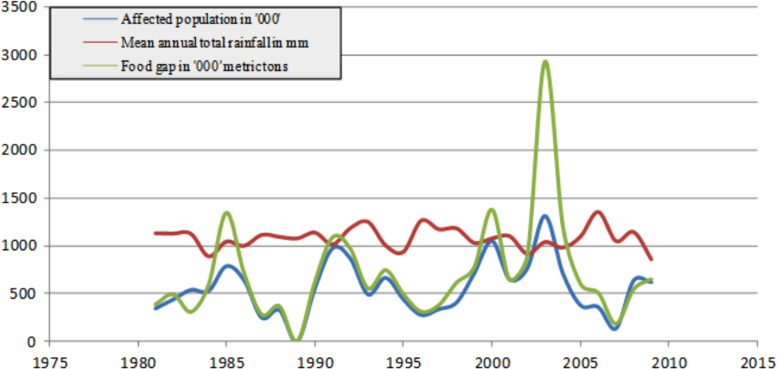
Association between rainfall pattern and food gap.*Source*: Computed based on raw data from NMA and CSA of Ethiopia.

**Fig. 17:**
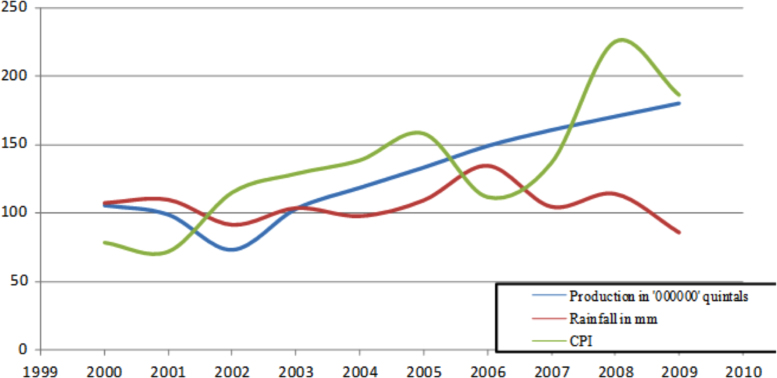
Association between rainfalls pattern, food production and consumer price index /CPI/.*Source*: Computed based on raw data from NMA and CSA of Ethiopia.

**Table 1:** Sample weather stations by PCI.

Variables	Weather Stations								
	Adama	A/Minch	Ciro	D/Dawa	Gode	Jigjiga	Moyale	Yabello	Ziway
Mean annual total rainfall in (mm)	859.9	889.7	929.0	611.0	236.00	698.2	723.3	⁎	748.9
PCI (%)	15.6	11.0	11.8	11.8	21.3	11.5	15.6	⁎	12.5

Note:

⁎ The data for Yabello Station is discontinuous.

**Table 2:** Long-term mean annual temperature and rainfall values of the stations.

Climatic Conditions	Meteorological sites							Average
	Adama	A/Minch	Ciro	D/Dawa	Gode	Jigjiga	Ziway	
Mean Max Temp. (°C)	28.5	30.4	28.0	32.0	34.8	27.5	27.0	29.7
Mean Min Temp. (°C)	14.7	17.3	14.0	19.1	23.5	11.3	14.0	16.3
Average Temp. (°C)	21.6	23.9	21.3	25.5	29.8	19.4	21.0	23.2
Mean annual rainfall (mm)	859.9	889.7	934.3	623.6	218.4	709.7	743.0	711.2

**Table 3:** Long-term seasonal average total rainfall values of the weather stations.

Meteorological sites and rainfall conditions		Seasons				
		Summer	Autumn	Winter	Spring	Annual
Adama	Amount of rainfall (mm)	518.5	133.6	59.2	171.7	883.0
	%	58.7	15.1	6.7	19.4	100.0
A/Minch	Amount of rainfall	153.3	311.9	116.9	354.6	936.7
	%	16.4	33.3	12.5	37.9	100.0
Ciro	Amount of rainfall	551.2	208.2	56.3	312.6	1128.3
	%	48.9	18.5	5.0	27.7	100.0
D/Dawa	Amount of rainfall	212.6	110.1	61.9	226.7	611.3
	%	34.8	18.0	10.1	37.1	100.0
Gode	Amount of rainfall	1.7	110.9	4.5	119.0	236.1
	%	0.7	47.0	1.9	50.4	100.0
Jigjiga	Amount of rainfall	253.7	157.5	52.2	234.8	698.2
	%	36.3	22.6	7.4	33.6	100.0
Ziway	Amount of rainfall	356.7	123.6	57.4	211.1	748.9
	%	47.6	16.5	7.7	28.2	100.0

## 2 Methods and materials

The unprocessed rainfall and temperature data were used to analyze the long-term temperature conditions, and the trend and variability of rainfall. Various mathematical procedures and techniques were used to analysis of climatic condition such as long-term monthly, seasonal and annual mean values, precipitation concentration index (PCI) and coefficient of rainfall variability. The PCI were applied in order to analyze the heterogeneity of precipitation and the relationship between variability and distribution of yearly precipitation. Moreover, the PCI was computed to look into the level of annual rainfall distribution (concentration or uniformity) all through the year. The PCI is described as [2], [3]:

$$PCI=100\;x\frac{\left(\sum {P_{i}}^{2}\right)}{{\left(\sum P_{i}\right)}^{2}}$$

where, _*Pi*_=is the monthly precipitation in month *i*

In this analysis, the PCI values of less than 10 represent a uniform distribution of rainfall (*i.e*. low precipitation concentration); the PCI values between 11 and 15 denote a moderate precipitation concentration; values from 16 to 20 denote irregular distribution and values above 20 represent a strong irregularity (i.e. high precipitation concentration) of precipitation distribution [1].

Rainfall variability over a period of time was analyzed by calculating the coefficients of variability of the rainfall values at different time scale. Hence, the coefficients of variability of annual and monthly rainfall for each station were calculated. Microsoft EXCEL and SPSS softwares were used to manage and analyze the data as well as to produce appropriate graphs for visualization of the trends of the variables.

According to de Luis et al. [1] classification, the PCI values between 11% and 15% denote a moderate precipitation concentration; values from 16% to 20% denote irregular distribution and values above 20% represent a strong irregularity of precipitation distribution. Based on this category, five of the sample sites (Arba-Minch, Ciro, Dire-Dawa, Jigjiga and Ziway) lie within moderate precipitation concentration; while two of them (Adama and Moyale) are characterized by irregularity of rainfall. The PCI value of Gode is over 20% denoting a strong irregularity of precipitation distribution in the area.

Fig. 12 (A–H) shows trends of spring (*belg*) rains of the stations.
