# Supplementary material for: Description of long-term climate data in Eastern and Southeastern Ethiopia
Source: Data Brief. 2017 Mar 18;12:26–36. doi: 10.1016/j.dib.2017.03.025 (PMC5567397; doi:10.1016/j.dib.2017.03.025)
Supplement: Supplementary file 1 — Supplementary material [file mmc1.docx]

**Disclosure statement**

No potential conflict of interest was reported by the author(s).
